# 

**Published:** 1888-08

**Authors:** 


					﻿io cts. a copy
AUGUST, 1888.
$ i .00 per year.
HAILS*
Jo\K\\l'
HEALTH
ESTABLISHED 1854
V°i- 35
cAl- 8. i
OFFICE: 206 BROADWAY, EVENING POST BUILDING, Boom 11. N. Y.
Entered as Second-class Matter at the Post-Office, New York.
				

